# Life expectancy inequalities in the elderly by socioeconomic status: evidence from Italy

**DOI:** 10.1186/s12963-018-0163-7

**Published:** 2018-04-12

**Authors:** Carlo Lallo, Michele Raitano

**Affiliations:** 1 Faculty of Education, Free University of Bozen, Via Ratisbona 16, 39042 Bressanone, Bolzano Italy; 2 Department of Economics and Law, Faculty of Economics, Sapienza University of Rome, Via del Castro Laurenziano 9, 00161 Rome, Italy

**Keywords:** Longevity, Health inequality, NDC pension systems, Cox model, Kaplan Meier simulations, Brass relational model

## Abstract

**Background:**

Life expectancy considerably increased in most developed countries during the twentieth century. However, the increase in longevity is neither uniform nor random across individuals belonging to various socioeconomic groups. From an economic policy perspective, the difference in mortality by socioeconomic conditions challenges the fairness of the social security systems. We focus on the case of Italy and aim at measuring differences in longevity at older ages by individuals belonging to different socioeconomic groups, also in order to assess the effective fairness of the Italian public pension system, which is based on a notional defined contribution (NDC) benefit computation formula, whose rules do not take into account individual heterogeneity in expected longevity.

**Methods:**

We use a longitudinal dataset that matches survey data on individual features recorded in the Italian module of the EU-SILC, with information on the whole working life and until death collected in the administrative archives managed by the Italian National Social Security Institute. In more detail, we follow until 2009 a sample of 11,281 individuals aged at least 60 in 2005. We use survival analysis and measure the influence of a number of events experienced in the labor market and individual characteristics on mortality. Furthermore, through Kaplan-Meier simulations of hypothetical social groups, adjusted by a Brass relational model, we estimate and compare differences in life expectancy of individuals belonging to different socioeconomic groups.

**Results:**

Our findings confirm that socioeconomic status strongly predicts life expectancy even in old age. All estimated models show that the prevalent type of working activity before retirement is significantly associated with the risk of death, even when controlling for dozens of variables as proxies of individual demographic and socioeconomic characteristics. The risk of death for self-employed individuals is 26% lower than that of employees, and life expectancy at 60 differs by five years between individuals with opposite socioeconomic statuses.

**Conclusions:**

Our study is the first that links results based on a micro survival analysis on subgroups of the elderly population with results related to the entire Italian population. The extreme differences in mortality risks by socioeconomic status found in our study confirm the existence of large health inequalities and strongly question the fairness of the Italian public pension system.

## Background

Life expectancy considerably increased in most developed countries during the twentieth century. According to United Nations (UN) data,^1^ average life expectancy at birth – for both sexes combined – was about 65 in the period 1950–1955 and is about 79 today in most developed countries (an area including Europe, North America, Japan, Australia, and New Zealand, according to the UN classification). In the first phase, increases in life expectancy were mainly due to a fall in infant and child mortality related to improvements in nutrition and public health that notably reduced the incidence of infectious diseases. Conversely, improvements in life expectancy after the 1970s began to be concentrated in those aged over 55, resulting in increases in longevity previously unmatched in human history [68]. In fact, further life expectancy at 60 was less than 17 years in the period 1950–1955, and it currently stands at about 23 years.

This increase in longevity, coupled with declining fertility rates, has led older people to represent an increasing share of population in most advanced countries, with a consequent aging of the population.

Originally, many authors were fascinated by the idea that improvements in medical care and healthier living conditions would give rise to an extended period of human life: a “third age” [38]. The third age should have been a time of self-fulfilment, free of disability and disease, in which people would have been free from the responsibilities of paid work and childcare to plan their lives and to pursue those plans.

However, the reality for governments is that they face far less promising outcomes in terms of the economic sustainability of the welfare state, because public spending for health care and pensions might greatly increase due to an aging population [25], unless the aging process is coupled with both a compression of older individuals’ morbidity and pension reforms that reduce the burden on public finances by curtailing benefits and tightening up retirement eligibility conditions.

The increase in longevity is neither uniform nor random across individuals belonging to various socioeconomic groups [23]. Indeed, the significant decrease in mortality in recent decades has not been equally distributed in the population, and the health gap between different groups of individuals has increased over time [24, 37, 60]. In other words, as pointed out by a vast literature (e.g., [29, 46]) health and longevity are completely unevenly distributed between individuals of different socioeconomic status, and these health gaps seem to be increasing over time, reinforcing the inequality.

From an economic policy perspective, the difference in mortality by socioeconomic conditions challenges the fairness of the social security system. Fairness is a complex concept related to subjective distributive justice values. For instance, following Nozick [55], some may argue that every outcome produced by free markets is fair if the individuals were entitled to the holdings they possessed before the market exchange, while, following Rawls [58], others may consider that compensatory policies to improve the incomes of the least well-off individuals should be always pursued. Likewise, as concerns the social security system and benefits received by the individuals, some may argue that – independent of the amount of contributions paid before retirement – a pension scheme that pays the same replacement rate (the ratio of pension benefit and final wage) to all individuals is fair, and others could even consider as fair a scheme that pays a flat-rate pension to all individuals.

In the pension economics literature, the concept of “actuarial fairness” has often been used recently as a benchmark [8]. Actuarial fairness implies that the internal rate of return that equals the actual value of contributions paid and pensions received over the life course (taking into account the probability of leaving a survivor benefit when this type of benefit exists) is the same for all individuals. In other words, if the same amount of contributions were paid during the working life by two individuals belonging to the same cohort of birth, pension wealth received during the life-course (i.e., the actual value of all benefits received since retirement up until death, or received by the survivor heir) should be the same. Likewise, if two individuals have paid a different amount of contributions, the internal rate of return on these contributions (i.e., the ratio of the actual values of pension wealth and paid contributions) should be the same.

Of course, one may argue that actuarial fairness does not imply an effective distributive justice if during their working life these individuals faced different opportunities to earn high wages and, hence, to pay high contributions – for instance, because they had different “circumstances” (i.e., factors for which the individuals are not responsible, such as gender, family background, or region of birth; [59]) or because poor health may have prevented them from achieving a successful career. According to this view, individuals should be compensated at retirement for their unequal outcomes/chances through a progressive redistribution that directs relatively more resources to those who paid lower contributions during the working life.

In what follows, we refer to the concept of actuarial fairness to assess the implications of the inequality of life expectancy on the fairness of a pension system (however, it should be noted that agreeing on more substantial concepts of distributive justice about the outcomes produced by the labor market would strengthen the arguments for progressive transfers toward the less favored workers after their retirement, for instance, when poor health worsened labor market outcomes during the active age).

Consider, for simplicity, the case of a pension scheme where the amount of monthly benefit is related to total contributions previously paid. If life expectancy differs between two individuals and the pension system does not take into account this difference, these individuals would receive a different pension wealth even if they paid the same contributions. Likewise, if they paid a different amount of contributions, the internal rate of return on these contributions – computed along the whole life – would differ between them. Indeed, in both cases, the individual with a lower longevity would be penalized. Thus, a social security system that computes pension benefits only according to the accrual of paid contributions – without taking into account differences in expected longevity – would not be actuarially fair and would then redistribute from those living less to those living more.

As a consequence, if people with a better socioeconomic status and higher income live longer, they will have a relatively higher pension wealth (with respect to the accumulated amount of contributions) during their lifetimes compared to people with lower socioeconomic status and incomes and a higher risk of death [10, 11, 15, 52]. Thus, without an appropriate compensation – concerning, for instance, retirement ages related to health conditions (a good predictor of longevity), allowing less advantaged individuals to receive a pension earlier and then to accumulate a higher pension wealth over the life cycle, or progressive pension benefit formulas that relatively favor those who paid less contributions and have (on average) a lower expected longevity – social security systems risk engendering a regressive redistribution over the course of an individual’s life (i.e., from the less well-off to the more well-off individuals), and this kind of redistribution clearly clashes with both actuarial fairness and more substantial concepts of distributive justice.^2^

Issues related to social differentiation in longevity are crucial in notional defined contribution (NDC) pension systems [57], i.e., in pay as you go public systems where benefits depend on the accrual of contributions paid during the working life and annuities are computed taking into account expected longevity at retirement (i.e., the number of years that a pension is expected to be paid). Indeed, if benefits are computed without differentiating longevity rates by individual characteristics – for instance, merely considering the average longevity at various ages – the pension system redistributes from those groups with a lower longevity to those with a higher longevity and actuarial fairness is not achieved. Furthermore, if – as recently established in some European Union (EU) countries^3^ – retirement age rises when average life expectancy of a population increases, independent of individual health status and expected longevity, a further disadvantage in terms of the relative share of life expectancy spent working might emerge – thus penalizing those coming from a worse socioeconomic condition.

A NDC pension system has been introduced in some countries around the world in recent decades – in Italy, Sweden, Poland, and Latvia within the EU – mostly because its technical characteristics allow policymakers to automatically control pension spending when longevity increases and the population gets older [33].

In Italy, the NDC system was introduced in 1995 [35]. For those who started to work in 1996 or later, the value of their future pension benefit will depend on the accrual of contributions paid during their whole working life. This accumulated amount is converted into an annuity at retirement using “transformation coefficients,” which are parameters based on the average life expectancy at a certain age of those resident in Italy.^4^ Transformation coefficients are updated every two years in order to take into account changes in longevity rates and are based on the average unisex life expectancy (also considering the probability of leaving a survivor’s pension) – i.e., they do not differ by gender or by other individual characteristics, such as an individual’s health or socioeconomic status (possible predictors of the expected individual longevity). Furthermore, the 2009–2010 reforms established that all requirements for having access to old-age pensions or early retirement benefits will be automatically updated to reflect changes in life expectancy at 65. Therefore, expected longevity has become a crucial feature of the Italian pension system.

As mentioned, the Italian NDC rules compute the value of pensions according to average life expectancy; hence, every non-causal deviation from that average (e.g., favoring some groups of individuals, for instance managers compared to blue-collar workers) might currently be bringing about redistribution of lifetime pension wealth – assessed relative to the total amount of contributions paid during the working life – from groups living less to groups living longer.

This article focuses on the case of Italy and aims at measuring differences in longevity at older ages by individuals belonging to different socioeconomic groups in order to assess the effective fairness of a social security system that, even if based on actuarial rules as concerns the link between pensions and the total contributions paid during the working life, does not take into account individual heterogeneity in expected longevity.

Several authors have shown that in Italy, as in most cases abroad, there is a significant correlation among socioeconomic condition, health, and mortality (e.g., [16]). However, despite several efforts to investigate mortality differences by socioeconomic status at older ages in Italy, the available evidence is not exhaustive due to limits in the available dataset. Furthermore, it should be emphasized that research in the international literature on the link between socioeconomic status and longevity in the elderly is more recent and less extensive than the research that has focused on the working-age population. Furthermore, as discussed below, the findings from studies on health inequality that have focused on the elderly are sometimes contradictory.

This article is an advance in respect to the current literature on longevity gaps by socioeconomic status at older ages in Italy. We use an innovative longitudinal dataset, called AD-SILC, that matches survey data on individual socioeconomic features recorded in the Italian module of the EU-SILC (European Union Statistics on Income and Living Conditions), with information on the whole working life and until death collected in the administrative archives managed by the Italian National Social Security Institute (INPS).

As clarified in the next sections, the AD-SILC dataset overcomes the limits of data sources previously used by those who have analyzed socioeconomic determinants of health and allows us to focus on the elderly population and estimate differences in life expectancies between the various socioeconomic groups. In more detail, we use survival analysis and measure the influence of a number of events experienced in the labor market and individual characteristics on mortality. Furthermore, by using Kaplan-Meier simulations of hypothetical social groups, adjusted by a Brass relational model, we estimate differences in life expectancy according to socioeconomic status for those aged over 60. We then compare life expectancies of individuals belonging to different socioeconomic groups. Hence, to the best of our knowledge, our study is the first that links results based on a micro survival analysis on subgroups of the elderly population with results related to the entire Italian population.

### Related literature

A vast and growing literature shows that dramatic differences in health conditions and in longevity between individuals characterized by different socioeconomic conditions have emerged across the globe (e.g., [46, 69]).

In an effort to explain such a divide, many epidemiologic investigations on differential mortality take a “lifestyle” approach, focusing on individual choices. People who smoke, drink alcohol, are overweight, or engage in dangerous sexual behaviours have a higher risk of numerous diseases than others [22, 56], and all these dangerous behaviors are not randomly distributed within the population but tend to concentrate in the lowest social classes [31, 42, 67]. Moreover, the effects on health of these dangerous behaviors also differ according to socioeconomic status, being stronger for blue-collar workers and low-paid clerks than for managers, employers, and professionals [14]. In addition, those individuals who have suffered an economic, social, or physical disadvantage in the past are more at risk of further damage of greater intensity in the future, a phenomenon known as the “chain of disadvantages” because each negative event amplifies the negative effect on quality of life, health, and survivorship [7, 21].

As pointed out by Marmot [45], the social divide in health is a remarkably widespread phenomenon and is not confined to those in poverty. It involves all of society, from the top to the bottom, with poorer health conditions emerging at every step in the social hierarchy. Therefore, the social divide takes on the characteristics of a social gradient.

Many studies have confirmed the existence of this social gradient, using different proxies of individual “socioeconomic position,” such as education, occupation, or income,^5^ and some studies have also shown an increasing trend in the social divide (e.g., [24, 39, 44]). Specifically, in developed countries the type of job plays a fundamental role in the social gradient of health: employment conditions determine adult socioeconomic status (i.e., their relational network and social environment) and has a direct effect on psycho-physic health [49].^6^ The literature has identified two main mechanisms through which the work environment directly influences individual health: the “demand-control” model [4, 36] and the “effort-reward” model [61, 66]. Both models suggest negative effects on health for employees and positive effects for managers, employers, and the self-employed, mainly due to the different typologies of stress they face and to the alienation of results [14].

Initially, research into the relationships between socioeconomic position and health was primarily focused on the working-age population. Analyses of the socioeconomic determinants of health and survival at older ages are more recent, and the results are sometimes contradictory: some longitudinal and cross-sectional studies [13, 53, 65] have shown a strong association between the previous occupation and health and survival at older ages, while other studies did not find a statistically significant relationship [1, 19] or found that the association declines with age [20, 63]. Trying to disentangle such an apparent contradiction, Marmot and Shipley [48] argued that the occupational class may strongly influence pre-retirement mortality, whereas other socioeconomic determinants of health arise after retirement. However, most of the studies measured the occupational group as self-reported by the individuals in old age in a single moment. In contrast, both the life-course hypothesis and the chain of disadvantage theory emphasize the accumulation and interaction of advantages and disadvantages across the entire lifespan, as a sort of time “exposed to risk” [6].

As regards Italy, despite several efforts to investigate mortality differences by socioeconomic status at older ages, the available empirical evidence is still not exhaustive, due to limits in the available dataset.

A dataset based on the linkage of death events and the Italian census – where information on occupation and education of the deceased was recorded – was created by the Italian National Statistical Institute (ISTAT) in 1991, but it was not updated in the ensuing years. Therefore, other data sources have been used in order to estimate mortality by social class of the Italian elderly. Some studies [5, 18, 40] used the administrative archives collected by the Italian Social Security Institute (INPS), which, however, do not record important socioeconomic variables such as education or marital status and, in the versions delivered until a few years ago, did not record complete working histories. In order to overcome these shortcomings, a dataset merging INPS archives with death registries and census information has been developed, but this dataset only refers to the city of Turin or to the region of Piedmont, thus preventing scholars from extending the analyses to the entire Italian population.

Instead of relying on microdata, other scholars have developed analyses following a macro perspective, correlating average values in different geographical areas of death rates and socioeconomic and demographic variables of the local population [43, 51]. However, due to what is called the “ecological fallacy,” the aggregate-level relationship between socioeconomic status and the average level of mortality in specific areas may be quite different from the individual-level association between such variables.

## Methods

### Data

We use an innovative dataset, called “AD-SILC,” built by merging the IT-SILC 2005 cross-sectional sample (i.e., the Italian module of the 2005 wave of the European Union Statistics on Income and Living Conditions – EU-SILC) and the administrative longitudinal records provided by the Italian National Social Security Institute (INPS). In detail, the cross-sectional variables collected in IT-SILC 2005 have been enriched by the longitudinal social security records since the entry in the labor market up to 2009.

Social security records offer a comprehensive picture of the working career of employees in the private sector (employees in the public sector were not enrolled with INPS until 2011) and all categories of the self-employed (professionals, though, are excluded because they are not registered with INPS) as they report, on a yearly basis and for each working relationship, region of residence, gross earnings, working weeks, the type of working relationship (thus allowing us to precisely distinguish the various categories of employees and the self-employed) and for pensioners, the type of benefit received (i.e., old-age pension, social pension, or disability benefit).

AD-SILC is innovative in the Italian context for two reasons: first, because of its retrospective (until 2005, the year of the EU-SILC interview) and prospective (from 2005 up to 2009) longitudinal design; and second, for its variety of available socioeconomic variables, unmatched before in any Italian study on social determinants of survival in old age.

From one side, AD-SILC is a retrospective unbalanced panel where longitudinal time-variant information on individuals’ working/pension histories is collected and linked to socioeconomic characteristics recorded in 2005. From another side, AD-SILC is a prospective dataset, with a base year in 2005 and a four-year follow-up. Indeed, we follow the histories of those interviewed in 2005 up until December 31, 2009. Therefore, the structure of the dataset allows us to observe the possible date of death until the end of 2009 of individuals who responded to the IT-SILC survey in 2005. Therefore, AD-SILC allows us to analyze mortality risks in the period 2005–2009 according to individual socioeconomic characteristics and according to their previous working history.

**Fig. 1:**
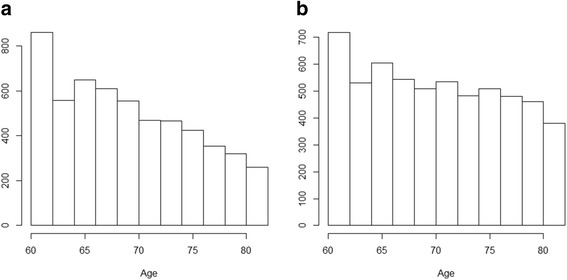
**a**. Age distribution of males at sample baseline, aged over 60. *Source: own elaborations on AD-SILC data.* **b**. Age distribution of females at sample baseline, aged over 60. *Source: own elaborations on AD-SILC data*

The AD-SILC panel size amounts to 1,162,045 observations, referring to 43,388 individuals interviewed by IT-SILC 2005 and recording at least once in INPS administrative archives. Since the aim of the present study is to estimate social determinants of mortality in old age, only individuals aged at least 60 on December 31, 2005, are included in the analysis (note that, according to OECD data,^7^ the effective retirement age was around 60 for males from the mid-1990s to the mid-2000s, while for females the effective retirement age was approximately one year lower). The sample used in the present study then amounts to 11,281 individuals: 5529 males and 5752 females. The age distribution in 2005 for males and females, respectively, is shown in Fig. 1a and b.

In order to carry out our analyses and to make the interpretation of the estimated coefficients easier, time-variant variables regarding working conditions over the whole life course are collapsed into a time-invariant variable, summarizing the prevalent condition along the career, where the prevalent condition has been defined as that with the longest time span over the working career. For instance, if an individual worked 10 years as an employee and 30 years self-employed, he/she is identified as self-employed. Note that our definition of the prevalent condition is independent of the final status before retirement; in the previous example, the individual would have been identified as self-employed even if he/she had spent the final part of his/her career as an employee.

We choose to rely on time-invariant covariates synthesizing the career trajectory instead of using more comprehensive information about the whole career for two reasons: i) since individuals might have had very complex employment trajectories (e.g., some years as a farmer, then an unemployment spell, then some years as an employee, then self-employed) it is extremely complicated, and in some sense arbitrary, to exogenously define – or to endogenously identify, for instance by using the sequence analysis – a wider set of categories summarizing these trajectories (and also the interpretation of the main findings of the empirical analysis would become less clear than if we consider a simple set of prevalent working statuses); ii) mostly we consider working categories that are rather invariant over the individual career; indeed, our data show that the prevalent condition is a very good proxy of the whole working trajectory: on average, in our sample, 87% of the individuals spent at least four-fifths of their working career in the status that we consider as prevalent and only 1% spent less than half of their working life in the status we consider as prevalent.

Working condition is identified in AD-SILC through information from the pension fund that the individual contributed to before they retired. Italian workers pay pension contributions to different funds depending on their working category: specific funds exist for employees in the private sector and for various categories of the self-employed, i.e., craftsmen, shopkeepers, and farmers (as mentioned, we do not have information on employees in the public sector or on professionals). Furthermore, for private employees, INPS archives also record occupation, which is coded through a dummy variable – white-collar versus blue-collar. Henceforth, exploiting information on the prevalent working category and the prevalent occupation during the working life, we distinguish five occupational groups: blue-collar employees in the private sector; white-collar employees in the private sector; self-employed craftsmen; self-employed shopkeepers; and farmers. Table 1 recaps the content of variables used in the present study.

**Table 1:** Summary and descriptions of variables used in this article

Variable	Categories	Sample distribution (%)
Sex	Male	49.0
	Female	51.0
Marital status	Unmarried	7.9
	Married	64.8
	Separated	2.1
	Divorced	1.4
	Widowed	23.8
Education	At most primary	66.3
	Lower secondary	15.5
	At least upper secondary	18.2
Ability to make ends meet	Low	31.9
	Medium	61.7
	High	6.5
Former occupational group	Blue-collar employee	51.9
	White-collar employee	13.7
	Self-employed shopkeeper	9.3
	Self-employed craftsman	9.9
	Farmer	15.3
Type of pension	Old-age pension	67.5
	Disability	6.9
	Social disability	3.3
	Social pension	22.3
Geographical area of residence	North-West	19.2
	North-East	16.5
	Center	33.5
	South and Islands	30.8
Age	Registered in 2005 and in 2009 (or in the death date)	
Date of death	Date of death is censored at December 31, 2009	

Source: elaborations on AD-SILC data

Exploiting information recorded in INPS archives, we also distinguish individuals according to the type of pension benefits received, which is strictly linked to their previous working history. We distinguish four categories of pension benefits: old-age pensions (pensions based on previous work contributions); disability pensions (paid to those injured at work and unable to continue working); social disability pensions (paid to the disabled, independent of the source of their disability); and social pensions (where we group two means-tested benefits directed to poor pensioners, namely minimum pensions and social assistance benefits).

Therefore, the subgroups we consider are not representative of the whole Italian elderly population since we include neither professionals and employees in the public sector – two categories that are likely to be characterized by a relatively higher life expectancy – nor those individuals who never worked (often female housewives) or have always worked in the informal sector (and then do not receive an old-age or a minimum pension when retired) and are not entitled to a disability benefit.^8^

Finally, IT-SILC records information, observed in 2005 on educational attainments (coded through the ISCED classification and grouped into three categories: at most primary educated, lower secondary educated, and at least upper secondary educated) and marital status. To better capture permanent household economic well-being, we also include in our analysis the information provided by the subjective variable self-reported by individuals in 2005 on the “ability to make ends meet,” whose six responses are grouped into three categories – low, medium, and high ability – for simplicity.^9^

### Empirical strategy

As argued above, the main aim of the present study is to estimate whether life expectancy of older Italians differs by their socioeconomic status. In particular, we investigate the association between having belonged to a certain occupational group during the working career and longevity at older ages, controlling for other individual demographics (i.e., gender, age, and marital status) and socioeconomic characteristics (i.e., education and household subjective economic condition).

We carry out our estimates by using the Cox semi-parametric proportional hazards model, which is the best-suited estimation model for samples containing truncated and censored data [2, 17].^10^

Alternative models to be applied to longitudinal microdata might be a parametric model (for example with a defined survival curve such as a Gompertz) or an accelerated failure time model (AFT). We prefer to use the Cox model because we do not want to invoke parametric assumptions about the shape of the baseline hazard function. Even if there is strong evidence in literature about the shape of the hazard function with respect to old-age human hazard, relaxing any assumption we will obtain survival data free to adapt to the real Italian survival curve, as officially estimated by ISTAT on the entire Italian population, which is our last step in this study, as explained further. However, we also estimated an alternative model using a Gompertz parametrization of the survival curve, obtaining substantially the same results with respect to the variable of interest in this study (see the results of the alternative *Model 4b* in Table 9, Appendix 2). On the other hand, AFT does not consent to perform a proper analysis when several censored and truncated data are present in the sample [54].^11^

We assumed “age” as time of entry into and exit from the dataset, and we estimated the parameters of the model using a MLE, considering both left truncation and right censoring. Moreover, we applied the Efron [26] method to overcome the problem of presence of ties in the dataset.

Afterward, we estimate the baseline hazard and the baseline survival curve, which only depends on time, not considering cohort stratification. Finally, using the Kaplan-Meier estimator it is possible to simulate the survival curve of a hypothetical social group with a certain covariate profile [64].

Assuming the individual age as the time of entry into and exit from the dataset, the computation of simulated survival curves stops at age 86, due to the limited sample size (less than 100 observations) in our dataset of individuals alive and aged over 86 by December 31, 2009. Therefore, it is possible to compute life expectancy at 60, but we have to truncate life expectancy at age 86. This outcome is then useful for a comparison between social groups but is not comparable to the Italian life expectancy at 60 because of its truncation.

However, using the Brass relational model, which is often used in the presence of incomplete or untrustworthy survival curves [12, 70], and comparing the Italian survival curve as officially computed by the ISTAT with the Kaplan-Meier simulations, we can compute a complete survival curve of the hypothetical groups from age 60 to age 105.

Brass suggests the following logit and anti-logit transformations:

1 $$ {Y}_x^s=\frac{1}{2}\ast \mathit{\ln}\left[\frac{l_{(x)}^s}{1-{l}_{(x)}^s}\right];\kern0.5em {Y}_x=\frac{1}{2}\ast \mathit{\ln}\left[\frac{l_{(x)}}{1-{l}_{(x)}}\right] $$

2 $$ {l}_x^s=\frac{\exp \left(2{Y}_x^s\right)}{1+\exp \left(2{Y}_x^s\right)}=\frac{1}{1+\exp \left(-2{Y}_x^s\right)};\kern0.5em {l}_x=\frac{\exp \left(2{Y}_x\right)}{1+\exp \left(2{Y}_x\right)}=\frac{1}{1+\exp \left(-2{Y}_x\right)} $$

where ${\boldsymbol{l}}_{\left(\boldsymbol{x}\right)}^{\boldsymbol{s}}$ are the values of a “standard” survival curve (complete and trustworthy, in this case the official Italian curve by gender, certified by ISTAT) and ***l***_(***x***)_ are the values of the uncomplete survival curve (in this case, the Kaplan-Meier simulations for every group defined by a “*k*” covariates profile).

The model then supposes a linear relationship between the logit of every human survival curve, as expressed below:

3 $$ {Y}_x=\alpha +\beta \ast {Y}_x^S $$

where the values ${\boldsymbol{Y}}_{\boldsymbol{x}}^{\boldsymbol{S}}$ are the logit of the standard survival curve and the values ***Y***_***x***_ are the logit of the uncomplete Kaplan-Meier simulations and **α** and **β** are the parameters estimated by a linear regression.

Following this simulation methodology, we can compare life expectancies of individuals belonging to different socioeconomic groups. Therefore, our study represents the first link for Italy between results based on a micro survival analysis with results related to the entire Italian population.

## Results

### Validation of the sample and checks for the assumption of proportionality

**Fig. 2:**
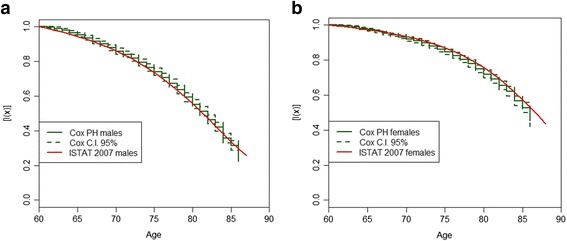
**a**. Validation of Cox model estimations on AD-SILC data, males. *Source: own elaborations on AD-SILC data.* **b**. Validation of Cox model estimations on AD-SILC data, females. *Source: own elaborations on AD-SILC data*

As a first outcome of our empirical analysis, we present a validation of both the sample and the methodology previously described. Figure 2a and b show, respectively for males and females, a comparison between the real survival curves of the Italian population and the curves simulated according to our data using the Kaplan-Meier estimator on Cox parameters. Results of the validation test confirm the goodness of both the AD-SILC sample and the methodology developed in the present study: the values of Italian survival curve by sex, as officially computed by the Italian National Statistical office, are always within 95% confidence interval (CI) of the simulated Kaplan-Meier curve.

Moreover, we performed a test to check the assumption of proportionality, analyzing the Schoenfeld scaled residuals [64]. From our analysis, the test results were not significant for any covariate except gender and disability pensions (*p*-value< 0.05). In particular, considering our preferred model where all sets of covariates are included and previous occupations are grouped in three categories (Model 4 in next section), the test results were not significant for both former job characteristics and self-perceived financial situation, which are the crucial variables of interest in this article.^12^ To control for potential bias due to the violation of the assumption of proportionality, we also ran alternative models [9, 34, 62], stratifying by gender and typology of pension (results of alternative *Model 4c* are reported in the Appendix 2, Table 10), but we obtained substantially the same results, both considering the magnitude of the effects and the statistical significance of parameters.

Therefore, taking into account the validation we did using official data on the Italian population, the results of the Shoenfeld residuals test and the estimates from alternative models, we consider that our model is an appropriate representation of data.

### Estimates of the association between mortality risk and individual features

We then show the results concerning the estimated association between individual characteristics – our main focus is on the previous occupation category – and mortality risk obtained by using the Cox semi-parametric model (Tables 2, 3, 4 and 5).

We start by estimating a first model (Model 1, Table 2) where we show the mortality hazard of different occupational groups, controlling for individual basic demographic variables such as gender, geographical area of residence, and marital status (age and cohort of birth are controlled by the structure of the model). Then, we estimate a second model (Model 2, Table 3), adding the type of pension benefit to the control variables, and a third model, where further socioeconomic characteristics are added to the regressors (namely, education and household socioeconomic conditions, as proxied by the subjective answer to the question about “ability to make ends meet”; Model 3, Table 4).

**Table 2:** Association between mortality risk and basic demographic characteristics

	Coefficient	Exp(coef)	S.E.	Pr(>|z|)
Female	−0.4769***	0.6207	0.0774	< 0.0001
Unmarried	0.2570**	1.2931	0.1127	0.02252
Separated	−0.0966	0.9079	0.2555	0.70528
Divorced	0.4272	1.5329	0.2662	0.10851
Widowed	0.1649*	1.1792	0.0878	0.06035
North-East	0.2150**	1.2398	0.0954	0.02418
North-West	0.2095**	1.2331	0.0914	0.02189
South & Islands	0.2177***	1.2432	0.0817	0.00769
White-collar employee	0.0433	1.0442	0.0954	0.65029
Self-employed craftsman	−0.4104***	0.6634	0.1197	0.00061
Self-employed shopkeeper	−0.8012***	0.4488	0.1357	< 0.0001
Farmer	−0.3800***	0.6839	0.0938	< 0.0001

*Model 1*

Reference categories are: “Male” for sex, “Married” for marital status, “Center” for geographical area of residence and “Blue-collar employee” for former occupational status. Significance legend: (***) = 99%; (**) = 95%; (*) = 90%. Source: elaborations on AD-SILC data

**Table 3:** Association between mortality risk, demographic characteristics, and typology of pension

	Coefficient	Exp(coef)	S.E.	Pr(>|z|)
Female	−0.5610***	0.5706	0.0791	< 0.0001
Unmarried	0.1646	1.1789	0.1149	0.1522
Separated	−0.2353	0.7904	0.2647	0.3740
Divorced	0.4321	1.5405	0.2717	0.1117
Widowed	0.1108	1.1172	0.0939	0.2379
North-East	0.2002**	1.2216	0.0969	0.0389
North-West	0.1428	1.1535	0.0933	0.1261
South & Islands	0.0330	1.0335	0.0840	0.6945
White-collar employee	0.1117	1.1182	0.0982	0.2553
Self-employed craftsman	−0.1601	0.8520	0.1215	0.1876
Self-employed shopkeeper	−0.4489***	0.6383	0.1402	0.0014
Farmer	−0.1830*	0.8328	0.0956	0.0557
Disability pension	0.4183***	1.5194	0.1038	< 0.0001
Social disability pension	1.3407***	3.8218	0.1283	< 0.0001
Social pension	0.1217	1.1294	0.0993	0.2205

*Model 2*

Reference categories are: “Male” for sex, “Married” for marital status, “Center” for geographical area of residence and “Blue-collar employee” for former occupational status, “Old-age pension” for type of pension. Significance legend: (***) = 99%; (**) = 95%; (*) = 90%. Likelihood Ratio Test between models 1 and 2 = 701.812 on 3 degree of freedom, p-value < 0.005. Source: elaborations on AD-SILC data

**Table 4:** Association between mortality risk, demographic characteristics, typology of pension, and socioeconomic characteristics

	Coefficient	Exp(coef)	S.E.	Pr(>|z|)
Female	− 0.5652 ***	0.5683	0.0805	< 0.0001
Unmarried	0.1422	1.1529	0.1169	0.22358
Separated	−0.2755	0.7592	0.2662	0.30068
Divorced	0.4254	1.5302	0.2715	0.11712
Widowed	0.0830	1.0865	0.0954	0.38447
North-East	0.2256 **	1.2531	0.0974	0.02061
North-West	0.1850 *	1.2032	0.0948	0.05109
South & Islands	0.0055	1.0055	0.0863	0.94919
White-collar employee	0.1558	1.1686	0.1067	0.14425
Self-employed craftsman	−0.1575	0.8542	0.1224	0.19819
Self-employed shopkeeper	−0.4083***	0.6648	0.1408	0.00374
Farmer	−0.1618*	0.8506	0.0973	0.09646
Disability pension	0.3862***	1.47132	0.10647	< 0.0001
Social disability pension	1.3323***	3.78957	0.13104	< 0.0001
Social pension	0.1136	1.12025	0.10053	0.25869
Edu. Lower secondary	−0.22983**	0.79467	0.10740	0.03236
Edu. Upper secondary or more	0.07138	1.07399	0.10182	0.48325
Medium ab. to make ends meet	−0.18922***	0.82760	0.07080	0.00753
High ab. to make ends meet	−0.60094***	0.54830	0.17771	0.00072

Reference categories are: “Male” for sex, “Married” for marital status, “Center” for geographical area of residence and “Blue-collar employee” for former occupational status, “Old-age pension” for type of pension, “Primary or none” for education level, “Low” for ability to make ends meet. Significance legend: (***) = 99%; (**) = 95%; (*) = 90%. Likelihood Ratio Test between models 2 and 3 = 260.894 on 4 degree of freedom, p-value < 0.005. Source: elaborations on AD-SILC data

All models clearly show that those who were previously self-employed – as a shopkeeper or a farmer – are characterized by statistically significant lower mortality risks when elderly than those previously employed as blue-collar workers. Conversely, mortality risks when elderly for former white-collar employees are not significantly different with respect to former blue-collar employees (note that results are substantially the same when setting former white-collar employees as the reference group of our estimates). Likewise, the difference in mortality risks between those self-employed as craftsmen and employees is only statistically significant in the first model. Moreover, we find no significant difference in mortality risks between former self-employed shopkeepers and craftsmen.

According to these results, and for the sake of simplicity in order to run computations of the following sections, we have aggregated blue-collar and white-collar workers into a single “omitted” category for employees, and shopkeepers and craftsmen into a single category for the self-employed (Model 4, Table 5). As expected, the main findings on the link between mortality risks and previous working condition do not change.

**Table 5:** Association between mortality risk, demographic characteristics, typology of pension, and socioeconomic characteristics

	Coefficient	Exp(coef)	S.E.	Pr(>|z|)
Female	−0.5764***	0.56190	0.08019	< 0.0001
Unmarried	0.1407	1.15112	0.11686	0.22847
Separated	−0.2697	0.76359	0.26621	0.31098
Divorced	0.4491*	1.56684	0.27071	0.09715
Widowed	0.0799	1.08317	0.09542	0.40245
North-East	0.2201**	1.24617	0.09740	0.02385
North-West	0.1871**	1.20568	0.09480	0.04849
South & Islands	0.0031	1.00307	0.08629	0.97166
Self-employed	−0.3011***	0.73999	0.09546	0.00161
Farmer	−0.1809*	0.83454	0.09605	0.05968
Disability pension	0.3862***	1.47132	0.10647	< 0.0001
Social disability pension	1.3323***	3.78957	0.13104	< 0.0001
Social pension	0.1136	1.12025	0.10053	0.25869
Edu. Lower secondary	−0.22983**	0.79467	0.10740	0.03236
Edu. Upper secondary or more	0.07138	1.07399	0.10182	0.48325
Medium ab. to make ends meet	−0.18922***	0.82760	0.07080	0.00753
High ab. to make ends meet	−0.60094***	0.54830	0.17771	0.00072

Aggregated former occupational groups. *Model 4*

Reference categories are: “Male” for sex, “Married” for marital status, “Center” for geographical area of residence and “Employee (blue+white collar)” for former occupational status, “Old-age pension” for type of pension, “Primary or none” for education level, “low” for household financial situation. Significance legend: (***) = 99%; (**) = 95%; (*) = 90%. Likelihood Ratio Test between models 4 and 3 = 0.21 on 2 degrees of freedom, p-value not significant. Source: elaborations on AD-SILC data

However, the type of pension benefit might be related to health, since individuals in poor health could receive a disability benefit or could have worked only rarely and are then entitled to receive only a social pension. To deal with this issue and partially take into account a possible reverse causality between poor health and the type of working career, as a robustness check we also ran our estimates excluding from the sample those who receive a social pension or both types of disability benefits (Tables 11–12 in Appendix 2). Our main results about a significantly different mortality between self-employed and employees is confirmed also by these robustness checks.

Apart from the link between occupation and mortality, which is the main focus of this article, it is interesting to observe the estimated association between mortality risks and the control variables included in our regressions.

As clearly expected, males and the disabled (i.e., those receiving a disability pension) are characterized by a significantly much higher risk of death, as evident in looking at the estimated mortality hazards in all models. When controlling for the typology of pension benefit and socioeconomic conditions, marital status does not show a significant association with mortality (except for the divorced, whose *p* value is close to 10%). Furthermore, apart from the first estimated model, those living in the North are characterized by a higher mortality risk compared to those living in the Center, while no significant differences between Central and Southern residents emerge.

As expected, the typology of pension benefit has a clear association with mortality hazards of retired Italians. The disabled suffer a clearly higher mortality hazard with respect to those who receive a standard pension benefit (mortality hazard is 50% and 280% higher for those permanently injured at work and for the disabled, respectively, compared to those who receive an old-age pension), and these results also hold when controlling for further socioeconomic characteristics.

With respect to socioeconomic controls (Models 3 and 4), as expected, the better the household economic condition (captured by the “ability to make ends meet” variable), the lower the mortality hazard. The mortality hazard of those who report they make ends meet easily or very easily (the “high” group) is 45% lower than the hazard characterizing those who report making ends meet with great difficulty and with difficulty. Likewise, those in the “medium” group show a hazard that is around 20% lower than those belonging to the “low” group.

Conversely, less clear findings emerge for educational level, controlling for an individual’s occupation, type of pension, and household economic condition (variables that are, however, clearly correlated to education). Indeed, compared to those having attained at most a primary education, a significantly lower mortality characterizes those with a lower secondary education, while no further advantage – on top of those mediated by having achieved a better occupation, type of pension, and household economic status –characterize those with an upper secondary or a tertiary education.

Finally, as the main finding of our estimates, it must be pointed out that the prevalent type of working activity before retirement is strongly associated with the risk of death, even when we control for dozens of variables as proxies of individual demographic and socioeconomic characteristics. Aggregating working categories, the risk of death for self-employed individuals is 26% lower than the risk for employees and, likewise, farmers’ risk of death is 17% lower than that of employees.

### Simulation of survival curves by individual characteristics

As a final outcome, we use Kaplan-Meier simulations of the survival curves of different groups of individuals aged over 60 (as already explained, these simulations are truncated at age 86 due to problems in mortality estimation of groups with a limited sample size; in our sample less than 100 individuals are aged over 85, see Fig. 1a and b), distinguishing individuals in various socioeconomic groups according to the values of relevant covariates.

We simulate nine hypothetical social groups combining three different categories of former occupation groups (employees, self-employed, and farmers) and three levels of ability to make ends meet (high, medium, low) and compute their life expectancy (Table 6, where we consider married males, old-age retired, living in the center of Italy, with a primary education). Therefore, for example, we call “Low Employee” a social group that combines the former occupational class “employee” and a “low” ability to make ends meet.

**Table 6:** Life expectancies at 60 truncated at 86 for different simulated social groups (95% confidence intervals in parentheses)

Household wealth	Former occupational class								
	Employees			Farmers			Self-employed		
	*(LCI)*	Estimated	*(UCI)*	*(LCI)*	Estimated	*(UCI)*	*(LCI)*	Estimated	*(UCI)*
Low	*(18.43)*	19.42	*(20.51)*	*(19.22)*	20.38	*(21.7)*	*(19.75)*	20.84	*(22.05)*
Medium	*(19.67)*	20.51	*(21.43)*	*(20.39)*	21.39	*(22.48)*	*(20.85)*	21.79	*(22.81)*
High	*(20.70)*	22.08	*(23.65)*	*(21.47)*	22.79	*(24.29)*	*(21.87)*	23.11	*(24.51)*

Categories of control variables are set as follows: male, married, resident in the center, old-age retired. Source: elaborations on AD-SILC data

**Fig. 3:**
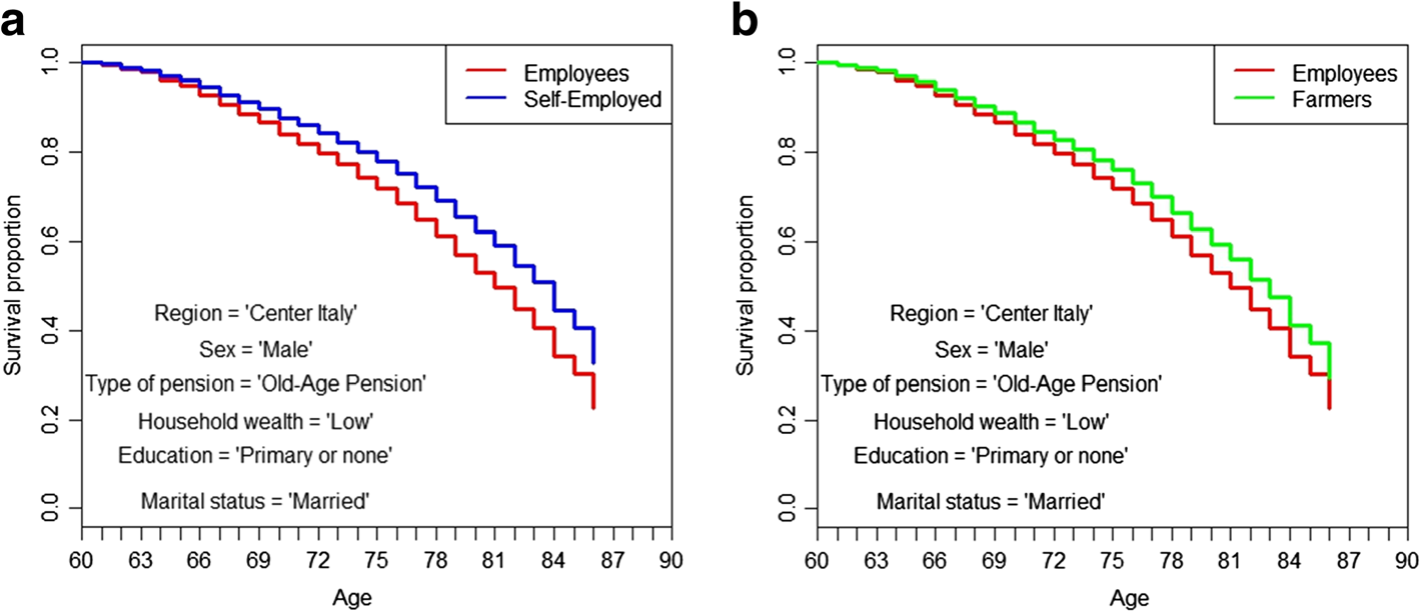
**a**. Kaplan-Meier simulated survival curves truncated at 86, employees versus self-employed. *Source: own elaborations on AD-SILC data.* **b**. Kaplan-Meier simulated survival curves truncated at 86, employees versus farmers. *Source: own elaborations on AD-SILC data*

We then show Kaplan-Meier simulated survival curves from age 60 to age 86 by occupation, for married males, old-age retired, living in the center of Italy, with a primary education and a low level of household wealth (Fig. 3a and b).

As already evident in the estimation results (Tables 2, 3, 4 and 5), the relation between former occupational class and mortality at older age does not disappear when controlling for financial situation (and other demographic variables).

According to these simulations, the self-employed have the highest life expectancy, and their life expectancy is 1.5 years higher than that of employees (Table 6). Moreover, the life expectancy of former employees with a medium level of ability to make ends meet, i.e., the “Medium Employee,” barely reaches the life expectancy of the “Low Self-Employed” (Table 6). Also, farmers have a higher life expectancy than employees, even though the divide is narrower (1 year, see Table 6).

**Fig. 4:**
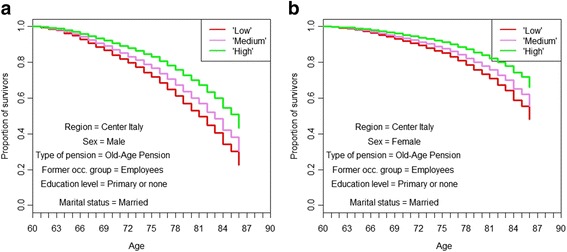
**a**. Kaplan-Meier simulated survival curves for different levels of household wealth, males. *Source: own elaborations on AD-SILC data.* **b**. Kaplan-Meier simulated survival curves for different levels of household wealth, females. *Source: own elaborations on AD-SILC data*

As expected, economic conditions are clearly related to survival (Fig. 4a and b). All other variables being equal, the gap in life expectancy between the opposite positions is almost three years for men and almost two for women.

**Fig. 5:**
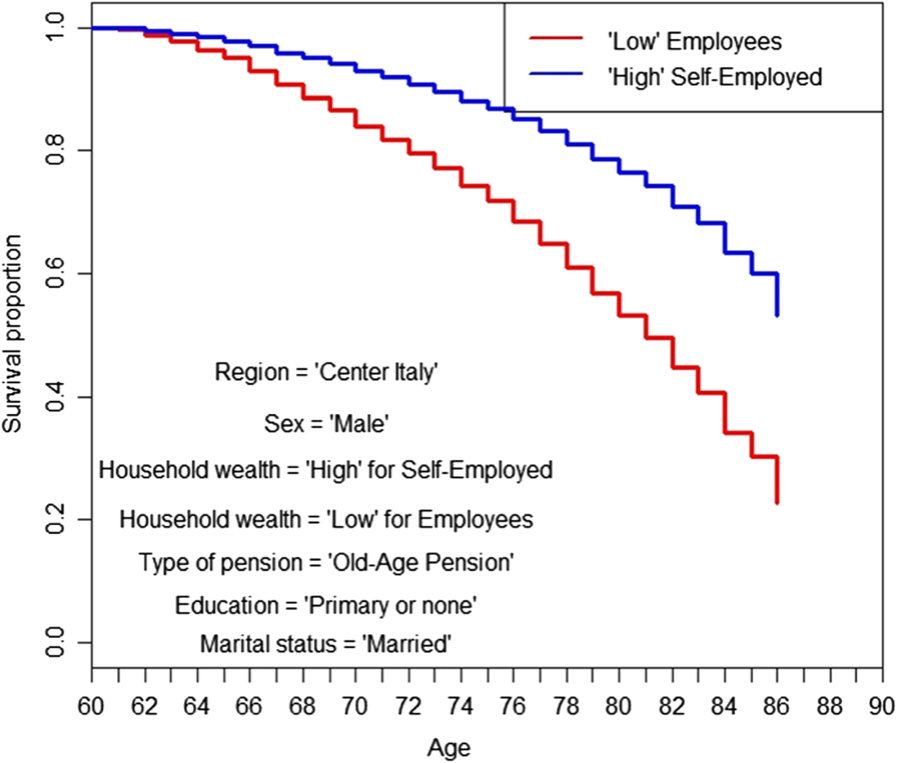
Kaplan-Meier simulated survival curves, employees with a low level of household wealth versus self-employed with a high level of household wealth. *Source: own elaborations on AD-SILC data*

Combining the type of prevalent previous working activity and the self-reported economic condition of households, differences increase exponentially, as shown by the simulated Kaplan-Meier survival curves in Fig. 5. Indeed, the difference in life expectancy at 60 truncated at 86, between a “low employee” and “high self-employed,” is about 3.5 years (see Table 6).

However, as already explained, these life expectancies cannot be compared with the Italian national life expectancy at 60 because of the truncation of Kaplan-Meier survival curve at 86.

**Fig. 6:**
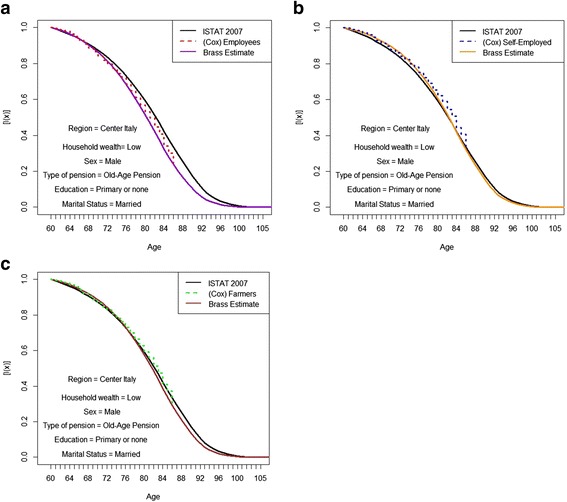
**a**. Kaplan-Meier simulated survival curves and Brass adjustments with Italian curve as reference. Employees with a low level of household wealth. *Source: own elaborations on AD-SILC data.* **b**. Kaplan-Meier simulated survival curves and Brass adjustments with Italian curve as reference. Self-employed with a low level of household wealth. S*ource: own elaborations on AD-SILC data.* **c**. Kaplan-Meier simulated survival curves and Brass adjustments with Italian curve as reference. Farmers with a low level of household wealth. *Source: own elaborations on AD-SILC data*

**Fig. 7:**
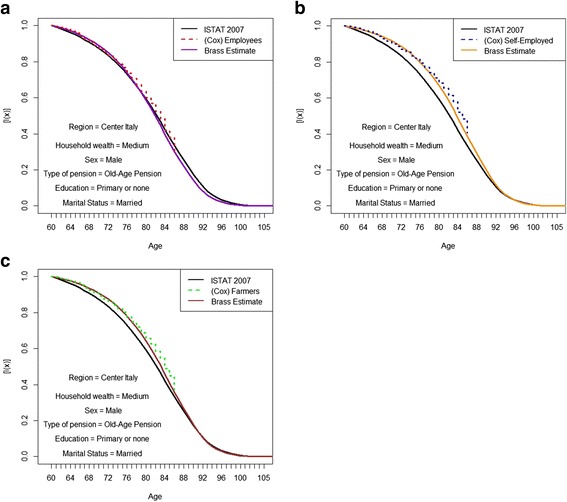
**a**. Kaplan-Meier simulated survival curves and Brass adjustments with Italian curve as reference. Employees with a medium level of household wealth. *Source: own elaborations on AD-SILC data.* **b**. Kaplan-Meier simulated survival curves and Brass adjustments with Italian curve as reference. Self-employed with a medium level of household wealth. *Source: own elaborations on AD-SILC data.* **c**. Kaplan-Meier simulated survival curves and Brass adjustments with Italian curve as reference. Farmers with a medium level of household wealth. *Source: own elaborations on AD-SILC data*

**Fig. 8:**
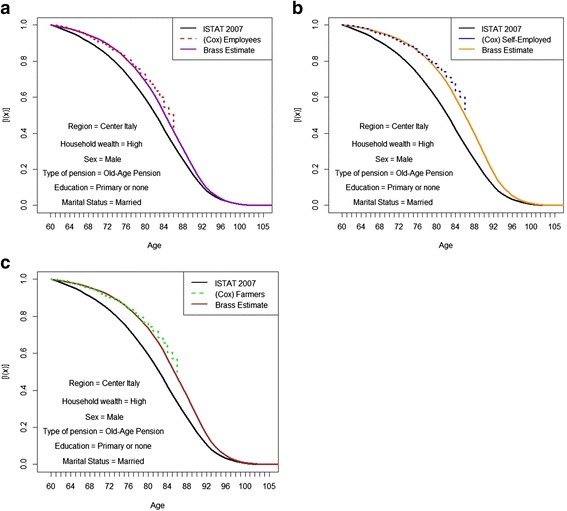
**a.** Kaplan-Meier simulated survival curves and Brass adjustments with Italian curve as reference. Employees with a high level of household wealth. *Source: own elaborations on AD-SILC data.* **b**. Kaplan-Meier simulated survival curves and Brass adjustments with Italian curve as reference. Self-employed with a high level of household wealth. *Source: own elaborations on AD-SILC data.* **c**. Kaplan-Meier simulated survival curves and Brass adjustments with Italian curve as reference. Farmers with a high level of household wealth. *Source: own elaborations on AD-SILC data*

In order to overcome this problem, Figs. 6, 7 and 8 show Brass estimations computed putting in relation the Italian survival curve, computed by ISTAT, and the Kaplan-Meier simulations. Table 7 compares life expectancies at 60 computed on the Brass adjusted survival curves of different simulated groups, with the Italian national life expectancy.

**Table 7:** Life expectancies at 60 for different simulated social groups adjusted by Brass (95% confidence intervals in parentheses) and for Italy

Household wealth	Former occupational class									Italy (2007)
	Employees			Farmers			Self-employed			
	*(LCI)*	Estimated	*(UCI)*	*(LCI)*	Estimated	*(UCI)*	*(LCI)*	Estimated	*(UCI)*	
Low	*(18.72)*	19.76	*(20.8)*	*(19.72)*	20.98	*(22.26)*	*(20.42)*	21.57	*(22.72)*	21.79
Medium	*(20.34)*	21.14	*(21.91)*	*(21.3)*	22.30	*(23.29)*	*(21.93)*	23.05	*(23.76)*	
High	*(21.67)*	23.28	*(24.99)*	*(22.74)*	24.35	*(26)*	*(23.33)*	24.87	*(26.43)*	

Categories of control variables are set as follows: male, married, resident in the center, old-age retired. Source: elaborations on AD-SILC data

Wide gaps between the average life expectancy for the Italian population and specific life expectancies for different population subgroups clearly emerge from our estimates and simulations. For instance, recalling our hypothetical groups, the other variables being constant, a “low employee” has a life expectancy two years shorter than the Italian value computed by ISTAT. In contrast, a “high self-employed” has a life expectancy three years longer than the average value for Italy (see Table 8).

**Table 8:** Social class and life expectancy at 60 of simulated socioeconomic groups (95% confidence intervals in parentheses)

*Social group*	*(LCI)*	*Life expectancy at 60*	*(UCI)*	*Difference from*	CI
				*Italian life expectancy (2007)*	
“High” self-employed	*(23.33)*	24.87	*(26.43)*	3.08	*[1.54] [4.64]*
“High” farmers	*(22.74)*	24.35	*(26)*	2.56	*[0.95] [4.21]*
“High” employees	*(21.67)*	23.28	*(24.99)*	1.49	*[−0.12] [3.2]*
“Medium” self-employed	*(21.93)*	22.87	*(23.76)*	1.08	*[0.14] [1.97]*
“Medium” farmers	*(21.3)*	22.3	*(23.29)*	0.51	*[−0.49] [1.5]*
“Low” self-employed	*(20.42)*	21.57	*(22.72)*	−0.22	*[−1.37] [0.93]*
“Medium” employees	*(20.34)*	21.14	*(21.19)*	−0.65	*[−1.45] [0.12]*
“Low” farmers	*(19.72)*	20.98	*(22.26)*	−0.81	*[−2.07] [0.47]*
“Low” employees	*(18.72)*	19.76	*(20.8)*	−2.03	*[−3.07] [−0.99]*

Categories of control variables are set as follows: male, married, resident in the center, old-age retired. Source: elaborations on AD-SILC data

Finally, the gap in life expectancy at 60 between opposite groups – i.e., those previously employees and currently in the poorest group (low-level households) and those previously self-employed belonging to the wealthiest group (high-level households) – even goes beyond five years.

## Discussion

This article has added new elements to the literature on health inequality in Italy.

First of all, the data and methodology used have allowed us to overcome many limits of previous studies. As frequently highlighted in the literature [6, 7] social determinants have a cumulative effect on life histories and should be analyzed in a longitudinal view rather than in a cross-sectional correlation model. Indeed, even though some studies based on INPS administrative data are currently available [5, 40], none of these studies have linked such administrative data with socioeconomic information from a survey sample, and none, before now, has used life expectancy to summarize the differential mortality.

Linkage of INPS administrative data with a sample survey is useful for many reasons. First of all, it has allowed us to include several demographic and socioeconomic controls. Second, retrospective INPS administrative data on the whole working and pension history allowed us to create a sort of “cumulative” index of social position, based on occupational position. This is fundamental to evaluate the association of occupational status with a variable proxy of the household economic condition (i.e., household subjective answers to the question about the “ability to make ends meet”). Moreover, while previous investigations have measured differences in mortality rates or mortality hazards, the present study uses life expectancy. This is very useful with respect to retirement policy evaluations, as it has allowed us to provide direct comparisons between the differing number of years expected to live after retirement by socioeconomic position based on occupation.

Second, the present study is part of the never-ending debate on the social determinants of health in older age [53], specifically on the relationships between socioeconomic groups, defined according to individual working history and mortality. Contrary to what was found by Amaducci et al. [1] for Italy, or by Dahl and Birkelund [19] for Norway, or by Marmot and Shipley for England (1996), but consistent with Luy et al. [41] for Italy and McMunn et al. [53] for Europe, our findings confirm that socioeconomic position, determined by (former) occupational group, strongly predicts life expectancy even in old age and after retirement.

The robustness and significance of our estimates is probably due to the specific method developed to define occupational groups, identified by the prevalent status over the whole working history as registered by INPS administrative archives, contrary to other studies, which used cross-sectional and/or self-reported information. Indeed, this confirms the life-course approach, both from the “accumulation of disadvantages” and the “chain of disadvantages” perspectives.

It is essential to highlight two aspects: the first is that the differences at old age are normally of lower intensity, since in previous years a “survivor effect” related to selective mortality operates. This means that such differences are even higher for life expectancy at birth. The second aspect is that our analysis does not cover the overall spectrum of society. Since we mainly focused on the previous occupation, we only included in the analysis those who have worked during their life. Furthermore, due to data limits, we also excluded professionals and employees in the public sector, two categories that are likely characterized by a higher longevity than average. Thus, our results can be considered as a sort of lower bound of the effective (very high) heterogeneity of life expectancy at older ages within Italian pensioners.

## Conclusions

The main goal of this study was to investigate and measure the magnitude of differences in life expectancy for the Italian elderly according to their socioeconomic status. A specific methodology, developed on different steps, has provided estimations on life expectancy of different social groups. Social gradient in survival has been proved, and the magnitude is remarkable: more than five years between individuals from opposite social positions. The results confirmed a social divide between occupational groups. In particular, the self-employed have an estimated advantage in life expectancy at 60 of almost two years, other socioeconomic and demographic conditions being equal. This confirms the persistence after retirement of the social determinants of mortality based on working relations, even after the “survivor effect.”

Combining working history and household economic condition – proxied by the answers to the question on “ability to make ends meet” – the estimated life expectancy of the lowest social group is characterized by a profile significantly below the national life expectancy (− 2 years), while, on the opposite side, the most advantaged group is characterized by a life expectancy clearly greater than the Italian level (+ 3 years).

This means that the Italian public pension system, which has a fixed legal age of retirement linked to the national average life expectancy and will compute annuities in the notional defined contribution pension system according to the average life expectancy at retirement age, is turning into a “regressive” redistribution system instead of guaranteeing an effective actuarial fairness. Therefore, from a policy perspective, even if finding exhaustive proxies of expected longevity is extremely complicated since longevity is affected by dozens of possible determinants, our findings strongly suggest that individual socioeconomic conditions – clearly affecting health and expected longevity – should be taken into account when designing insurance and pension schemes based on life expectancy.

## Appendix 1

### Methods

Cox model is generically defined by two factors:

4 $$ {h}_i(t)={h}_0(t)\ast \exp \left\{{k_i}^T\ast \beta\ \right\} $$

where ***h***_**0**_(***t***) is the baseline hazard, i.e., not specified and depends only on time, while $\mathbf{\exp}\left\{{{\boldsymbol{k}}_{\boldsymbol{i}}}^{\boldsymbol{T}}\ast \boldsymbol{\beta}\ \right\}$ are the fixed estimated effects, that depend only on the transposed matrix of covariates ***k***_***i***_^***T***^ and on the respective parameters ***β***, but are not affected by time.

The Partial Likelihood function, which has to be maximized, is built as follows:

5 $$ L={\prod}_{i=1}^n{\left(\frac{e^{x_i\beta }}{S^{(0)}\left(\beta, t\right)}\right)}^{\delta_i} $$

where:

- ***x***_***i***_ is an individual with the covariate profile “*i*” who experienced the death/censoring at time (*t*_*i*_)

$$ {\boldsymbol{\delta}}_{\boldsymbol{i}}=\left\{\begin{array}{c}0\to if\ an\ individual\ leaves\ the\ dataset\  at\  time\ \left({t}_i\right)\  due\  to\ censoring\\ {}1\to if\ an\ individual\ leaves\ the\ dataset\  at\  time\ \left({t}_i\right) due\  to\ death\kern2.5em \end{array}\right. $$

- ***S***^(**0**)^(***β***, ***t***) = $$ \sum \limits_{k=1}^n{Y}_k(t)\ {e}^{x_k\beta };\kern0.5em with\ {Y}_k(t)=I\ \left({L}_i<t\le {T}_i\right) $$

$$ {\boldsymbol{L}}_{\boldsymbol{i}}=\mathrm{age}\ \mathrm{of}\ \mathrm{entering}\ \mathrm{in}\ \mathrm{the}\ \mathrm{dataset} $$

$$ {\boldsymbol{T}}_{\boldsymbol{i}}=\mathrm{age}\ \mathrm{of}\ \mathrm{death}/\mathrm{censoring} $$

- **β** are the parameters to be estimated.

Assuming *age*, as the time of entry and exit from dataset (due to censoring – i.e., an individual still alive at the end of 2009 – or death), our dataset presents a contemporary entry and exit of individuals, due to data artefact. Indeed, even if INPS theoretically registers the exact date of death during a year, the administrative archives are checked for consistency every December 31 in order to correct measurement errors concerning the date of death. Because of the possible existence of these errors, we do not exploit specific date of death and consider the year of the unit of measurement of the age at death. Therefore, we make use of the likelihood function built by Efron [26], which is very well-suited to these situations. The function is formalized as follows:

6 $$ L={\prod}_{i=1}^I\frac{\prod_{j\in \mathfrak{D}\left({y}_{(j)}\right)}{e}^{x_z\beta }}{\prod_{v=1}^{\left|\mathfrak{D}\left({y}_{(j)}\right)\right|}\left[{\sum}_{i\in \mathfrak{R}\left({y}_{(j)}\right)}{e}^{x_z\beta }-\frac{v-1}{\left|\mathfrak{D}\left({y}_{(j)}\right)\right|}{\sum}_{j\in \mathfrak{D}\left({y}_{(j)}\right)}{e}^{x_z\beta}\right]} $$

where:

- ***y***_(***j***)_: is the time of exit of each group of individuals (Assuming 5 individuals that die at the following times: [1,1,3,3,3,3], *y*_(*j*)_ would assume values: *y*_(1)_ = 1 e *y*_(2)_ = 3)
- ***I***: number of individuals dying at time *y*_(*j*)_ (From the previous example: *I*_(1)_ = 2 e *I*_(2)_ = 4)
- ${\mathbf{\mathfrak{D}}}_{\left(\boldsymbol{y}\right)}$: is the “death set” of individuals dying at time *y.*
- ${\mathbf{\mathfrak{R}}}_{\left(\boldsymbol{y}\right)}$: is the “risk set,” all the individuals in the dataset at time *y*.
- ***x***_***z***_***β***: are all the individuals of dataset with their respective covariates *z* and parameters *β*.

Finally, we estimate the parameters following an iterative process described by Newton-Raphson. The iteration considers censored individuals still in the dataset just an instant before the exits due to death, in accordance with the administrative conventions of INPS.

Using the covariates previously presented, we specify the following estimation model stratified by cohort of birth (σ):

7 $$ {\mathrm{h}}_{\mathrm{i}}\left(\mathrm{t}\right)={{\mathrm{h}}_0}_0^{\upsigma}\left(\mathrm{t}\right)\ast \exp \left\{\begin{array}{c}\mathrm{sex}\ast {\upbeta}_1+{\mathrm{r}\mathrm{egion}}_{\mathrm{r}}\ast {\upbeta}_{\mathrm{r}}+{\mathrm{marital}\ \mathrm{status}}_{\mathrm{j}}\ast {\upbeta}_{\mathrm{j}}+\\ {}{\mathrm{education}\ \mathrm{level}}_{\mathrm{y}}\ast {\upbeta}_{\mathrm{y}}+\\ {}{\mathrm{h}\mathrm{ousehold}\ \mathrm{wealth}}_{\mathrm{k}}\ast {\upbeta}_{\mathrm{k}}+\\ {}\mathrm{prevalent}\ \mathrm{former}\ {\mathrm{occupational}\ \mathrm{class}}_{\mathrm{f}}\ast {\upbeta}_{\mathrm{f}}+\\ {}\mathrm{prevalent}\ \mathrm{type}\ {\mathrm{of}\ \mathrm{pension}}_{\mathrm{p}}\ast {\upbeta}_{\mathrm{p}}\ \end{array}\right\} $$

We estimate β parameters using the Cox model. Afterwards, we estimate the baseline hazard and the baseline survival curve, which only depends on time, not considering cohort stratification. Finally, using the Kaplan-Meier estimator it is possible to simulate the survival curve of a hypothetical social group with a certain covariate profile [64].

We assume that the baseline survival curve *S*_0_(*t*) has a jump only in the points *q* defined by times *t*_(1)_, *t*_(2)_, *t*_(*q*)_, …, *t*_(*Q*)_ when death events happen. However, due to left truncation, we estimate a conditional survival function. Defining (*l*_*i*_) as the left truncation age, parameters are estimated maximizing the likelihood function in [5].

8 $$ {\widehat{S}}_0(t)=\left\{\begin{array}{c}\ 1\kern35em if\ t<{l}_i\\ {}\\ {}{\prod}_{1\le j\le q}{\prod}_{i\in {\mathfrak{D}}_j}\left({S}_0{\left({t}_j\right)}^{\mathit{\exp}\left\{\widehat{\beta}{k}_j\right\}}-{S}_0{\left({t}_{\left(j+1\right)}\right)}^{\mathit{\exp}\left\{\widehat{\beta}{k}_j\right\}}\right){\prod}_{i\in {\mathfrak{C}}_j}{S}_0{\left({t}_{\left(j+1\right)}\right)}^{\mathit{\exp}\left\{\widehat{\beta}{k}_i\right\}}\kern0.5em if\ t\ge {l}_i\end{array}\right. $$

where:

- ${\mathbf{\mathfrak{D}}}_{\boldsymbol{j}}$: is the “death set” of individuals dying at time *t*_(*j*)_
- ${\mathbf{\mathfrak{C}}}_{\boldsymbol{j}}$: is the “censored set” of individuals censored at time *t*_(*j*)_

Considering *l*_*i*_ ≥ *t* and defining *α*_*j*_ as the conditioned survival probability at time *t*_(*j*)_:

$$ {\alpha}_j=P\left(T>{t}_{(j)}|\ T\ge {t}_j,k=0\right) $$

Consequentially:

9 $$ {S}_0(t)={\prod}_{j\mid {t}_j\le t}{\alpha}_j $$

The likelihood function becomes:

10 $$ L={\prod}_{1\le j\le q}\left({\prod}_{i\in {\mathfrak{D}}_j}\left(1-{\alpha}_j^{\mathit{\exp}\left\{\widehat{\beta}{k}_i\right\}}\right)\ {\prod}_{i\in \mathfrak{R}\left({t}_{(j)}\right)-{\mathfrak{D}}_j}{\alpha}_j^{\mathit{\exp}\left\{\widehat{\beta}{k}_i\right\}}\right) $$

Differentiating the logarithm of the likelihood function with respect to *α*_*j*_, we obtain the maximum likelihood estimation of *α*_*j*_, i.e., ${\widehat{\alpha}}_j$. However, it is impossible to do this analytically because of multiple exits of dataset for each time [26, 28]. Then we estimate the parameters through an iterative process described by Newton-Raphson.

Finally, the survival baseline curve is:

11 $$ {\widehat{\mathrm{S}}}_0(t)={\prod}_{j\mid {t}_j\le t}\widehat{\alpha_j} $$

The survival curve at each time “t” of the hypothetical social group “i” with covariates profile “k” is:

12 $$ {\widehat{\mathrm{S}}}_{\left(t,{i}_k\right)}={\left[{S}_0(t)\right]}^{e^{k\beta}} $$

## Appendix 2

### Additional Results

**Table 9:** Association between mortality risk, demographic characteristics, typology of pension, and socioeconomic characteristics

	Coefficient	Exp(coef)	*Exp(coef) Model 4*	SE	L.95% CI	U.95%CI
Female	−0.6888	0.5022	*0.5619*	0.079	0.43	0.59
Unmarried	0.1359	1.1455	*1.1511*	0.115	0.91	1.43
Separated	−0.1693	0.8443	*0.7636*	0.263	0.50	1.41
Divorced	0.5396	1.7154	*1.5668*	0.264	1.02	2.88
Widowed	0.1218	1.1296	*1.0832*	0.094	0.94	1.36
North-East	0.1857	1.2040	*1.2462*	0.097	1.00	1.45
North-West	0.1768	1.1934	*1.2057*	0.094	0.99	1.43
South & Islands	0.0220	1.0223	*1.0031*	0.085	0.87	1.21
Self-employed	−0.2723	0.7616	0.7400	0.094	0.63	0.92
Farmer	−0.1862	0.8301	0.8345	0.095	0.69	1.00
Disability pension	0.4091	1.5054	*1.4713*	0.104	1.23	1.85
Social disability pension	1.3329	3.7921	*3.7896*	0.127	2.95	4.87
Social pension	0.1026	1.1080	*1.1203*	0.099	0.91	1.35
Edu. lower secondary	−0.1564	0.8552	*0.7947*	0.106	0.70	1.05
Edu. upper secondary	0.0465	1.0476	*1.0740*	0.100	0.86	1.28
Medium ab. to make ends meet	−0.1949	0.8229	*0.8276*	0.070	0.72	0.94
High ab. to make ends meet	−0.5468	0.5788	*0.5483*	0.175	0.41	0.82

Gompertz parametric estimates (Model 4b) and comparison with Model 4 estimates (Exponential of coefficients)

Reference categories are: “Male” for sex, “Married” for marital status, “Center” for geographical area of residence, “Employee (blue+white collar)” for former occupational status, “Old-age pension” for type of pension, “Primary or none” for educational level, “Low ability” for household financial situation. Source: elaborations on AD-SILC data

**Table 10:** Association between mortality risk and socioeconomic characteristics

	Coefficient	Exp(coef)	SE	Pr(>|z|)
Unmarried	0.1380	1.1479	0.1244	0.2673
Separated	−0.1913	0.8259	0.2751	0.4869
Divorced	0.4657	1.5931	0.2861	0.1035
Widowed	0.0785	1.0817	0.1027	0.4444
North-East	0.2076**	1.2308	0.1018	0.0413
North-West	0.1930*	1.2129	0.0989	0.0509
South & Islands	0.0265	1.0269	0.0902	0.7688
Self-employed	−0.3066***	0.7360	0.1008	0.0024
Farmer	−0.2600**	0.7711	0.1025	0.0112
Edu. lower secondary	−0.2263**	0.7975	0.1126	0.0444
Edu. upper secondary	0.0951	1.0998	0.1051	0.3654
Medium ab. to make ends meet	−0.1691**	0.8444	0.0752	0.0244
High ab. to make ends meet	−0.575***	0.5626	0.1880	0.0022

Model stratified by cohorts, gender, and typology of pension. Model 4c

Reference categories are: “Married” for marital status, “Center” for geographical area of residence, “Employee (blue+white collar)” for former occupational status, “Primary or none” for educational level, “Low ability” for household financial situation. Significance legend: (***) = 99%; (**) = 95%; (*) = 90%. Source: elaborations on AD-SILC data

**Table 11:** Association between mortality risk and basic demographic characteristics, only old-age retired

	Coefficient	Exp(coef)	SE	Pr(>|z|)
Female	− 0.6001***	0.5487	0.10220	< 0.0001
Unmarried	0.1995	1.2208	0.14123	0.1578
Separated	−0.1529	0.8582	0.31492	0.6274
Divorced	0.4274	1.5332	0.33471	0.2017
Widowed	0.1562	1.1690	0.12822	0.2232
North-East	0.0897	1.0938	0.12045	0.4566
North-West	0.0589	1.0606	0.11443	0.6071
South & Islands	0.0519	1.0533	0.11059	0.6388
White-collar employee	0.13378	1.1432	0.11547	0.2470
Self-employed craftsman	−0.17558	0.8390	0.15306	0.2500
Self-employed shopkeeper	−0.31216**	0.7319	0.16198	0.0500
Farmer	−0.2215*	0.8013	0.13783	0.1000

*Model 1*

Reference categories are: “Male” for sex, “Married” for marital status, “Center” for geographical area of residence and “Blue-collar employee” for former occupational status. Significance legend: (***) = 99%; (**) = 95%; (*) = 90%. Source: elaborations on AD-SILC data

**Table 12:** Association between mortality risk, demographic characteristics, typology of pension, socioeconomic characteristics, only old-age retired

	Coefficient	Exp(coef)	SE	Pr(>|z|)
Female	−0.6040***	0.54662	0.10368	< 0.0001
Unmarried	0.1753	1.19158	0.14280	0.21965
Separated	−0.2369	0.78907	0.31744	0.45550
Divorced	0.4284	1.53479	0.33414	0.19981
Widowed	0.14497	1.15601	0.12955	0.26311
North-East	0.1237	1.13168	0.12075	0.30563
North-West	0.1046	1.11024	0.11561	0.36570
South & Islands	0.0224	1.02264	0.11301	0.84295
Self-employed	−0.2434**	0.78396	0.11567	0.03536
Farmer	−0.2273*	0.79665	0.13808	0.09967
Edu. lower secondary	0.1764	0.83825	0.12858	0.17001
Edu. upper secondary or more	0.1471	1.15852	0.11563	0.20317
Medium Ab. To make ends meet	−0.2008**	0.81807	0.09149	0.02817
High Ab. To make ends meet	−0.8598***	0.42327	0.23242	0.00022

Aggregated former occupational groups. *Model 4*

Reference categories are: “Male” for sex, “Married” for marital status, “Center” for geographical area of residence and “Employee (blue+white collar)” for former occupational status, “Primary or none” for education level, “low” for household financial situation. Significance legend: (***) = 99%; (**) = 95%; (*) = 90%. Source: elaborations on AD-SILC data

## Abbreviations

AFT: Accelerated failure time model
EU: European Union
EU-SILC: European Union Statistics on Income and Living Conditions
INPS: Italian National Social Security Institute
ISTAT: Italian National Statistical Institute
NDC: Notional defined contribution
UN: United Nations

## Footnotes

1 Data are available at https://esa.un.org/unpd/wpp/Download/Standard/Mortality.

2 Of course, longevity is related to many other factors beyond individual socioeconomic status. This is the reason why it is extremely complicated to include simple proxies of expected individual longevity in formulas computing pension benefits to avoid a regressive redistribution related to different mortality. However, what we are interested in here is to point out the possible implications for the actuarial fairness of a pension system – and the effective redistribution during the whole life between low- and high-income groups – when non-causal deviations from average life expectancy characterize those with different socioeconomic characteristics.

3 See European Commission [27].

4 Individuals already employed in 1995 will receive a pension partially based on the NDC formula [35].

5 See, among others, Blane [6]; Frijters [30]; Donkin et al. [23]; Marmot et al. [47]; WHO-CSDH [69]; Marmot and Wilkinson [50].

6 Issues of individual unobserved heterogeneity and reverse causality might bias the estimate of a causal link between working career and health and longevity [3, 32]. Indeed, if workers with greater health risks sort into more stressful jobs (unobserved heterogeneity) or health influences labor supply and career success (reverse causality), the estimated effects of the characteristics of working life on health may be spurious [32]. In a robustness check of our empirical analyses, we partially take into account the issue of reverse causality, also carrying out the estimates excluding from the sample individuals who do not fulfil the requirements to get an old-age pension (i.e., those receiving a disability benefit or a social pension).

7 See data available at http://www.oecd.org/els/emp/average-effective-age-of-retirement.htm.

8 Because the latter group is highly heterogeneous – for instance, both household-rich housewives and vulnerable individuals who have always worked in the informal sector are included – no clear expectations about the longevity of this group can be made.

9 The question on the IT-SILC questionnaire is: “A household may have different sources of income and more than one household member may contribute to it. Thinking of your household’s total monthly or weekly income, is your household able to make ends meet?” The respondent’s assessment is coded through six categories – with great difficulty, with difficulty, with some difficulty, fairly easily, easily, very easily. In this article we group these categories into three groups: low ability (with great difficulty and with difficulty), medium ability (with some difficulty and fairly easily), and high ability (easily and very easily).

10 Technical details of the Cox model when samples present truncated and censored data are shown in Appendix A.

11 Moreover, AFT requires a specified survival curve, thus preventing the obtaining of robust estimations.

12 Specifically, the *p*-values are as follows: 0.75 for “Self-employed”; 0.66 for “Farmers”; 0.39 for “Medium ability to make ends meet”; 0.63 for “High ability to make ends meet.”
